# Nonequilibrium fluctuations as a distinctive feature of weak localization

**DOI:** 10.1038/srep10705

**Published:** 2015-05-29

**Authors:** C. Barone, F. Romeo, S. Pagano, C. Attanasio, G. Carapella, C. Cirillo, A. Galdi, G. Grimaldi, A. Guarino, A. Leo, A. Nigro, P. Sabatino

**Affiliations:** 1 Dipartimento di Fisica “E.R. Caianiello”, Università di Salerno, I-84084 Fisciano, Salerno, Italy; 2 CNR-SPIN, UOS di Salerno, I-84084 Fisciano, Salerno, Italy; 3 Dipartimento di Ingegneria dell’Informazione, Ingegneria Elettrica e Matematica Applicata, Università di Salerno, I-84084 Fisciano, Salerno, Italy

## Abstract

Two-dimensional materials, such as graphene, topological insulators, and two-dimensional electron gases, represent a technological playground to develop coherent electronics. In these systems, quantum interference effects, and in particular weak localization, are likely to occur. These coherence effects are usually characterized by well-defined features in dc electrical transport, such as a resistivity increase and negative magnetoresistance below a crossover temperature. Recently, it has been shown that in magnetic and superconducting compounds, undergoing a weak-localization transition, a specific low-frequency 1/f noise occurs. An interpretation in terms of nonequilibrium universal conductance fluctuations has been given. The universality of this unusual electric noise mechanism has been here verified by detailed voltage-spectral density investigations on ultrathin copper films. The reported experimental results validate the proposed theoretical framework, and also provide an alternative methodology to detect weak-localization effects by using electric noise spectroscopy.

Perturbative corrections to the Drude model, due to the quantum mechanical nature of the electron, are collectively known as quantum transport phenomena. One of the most studied examples of these phenomena, arising from electron interference, is weak localization (WL)^1^. Recently, WL has been observed in innovative two-dimensional systems like graphene^2^, topological insulators^3^, and two-dimensional electron gases^4^, which are considered very important for the development of future quantum computing applications. This discovery has attracted the attention of the scientific community, increasing the interest on the topic of charge carriers localization effects.

A striking feature of WL is represented by a resistivity increase below a characteristic crossover temperature, usually occurring at low temperatures^5^. This behaviour has been found in ultrathin metallic films (Cu, Mg, Ag, Au, Pt, with thickness *t* < 20 nm)^6^; in alloys and composites such as Au-Pd, Cu-CuO, Al-Al_2_O_3_, Au_1−*x*_ Ge_*x*_, Nb_1−*x*_ Si_*x*_^6^; in doped semiconductors as Si:P^7^, or Ge:Sb^8^. Metallic oxides, being strongly correlated electronic systems, can show a crossover temperature at values higher than 30 K^9^,^10^. In two relevant cases of oxide conductors, peculiar electric noise properties have been related to WL and interpreted in terms of nonequilibrium universal conductance fluctuations^11^,^12^. In particular, it has been reported that the bias current plays a fundamental role when quantum interference effects become significantly evident, giving origin to an unusual linear dependence of the voltage-spectral density^12^. Noise spectroscopy has already demonstrated its potentialities by providing information on the dynamic behaviours and the kinetic processes of the charge carriers in several condensed matter systems^13^,^14^,^15^,^16^,^17^. However, a conclusive response on the strict connection between this type of fluctuations and WL can only be obtained by investigating simple metals, like copper, that have unambiguously shown the presence of quantum conduction mechanisms at low temperatures^18^,^19^,^20^.

In view of all these considerations, detailed dc electric- and magneto-transport measurements, as well as voltage-noise analysis, of ultrathin Cu samples are here reported and interpreted in the framework of nonequilibrium universal conductance fluctuations. Seven different films, with thickness from 7 to 200 nm, have been deposited by dc magnetron sputtering on Si(100) substrates. This choice of analyzing a wide thickness range is necessary in order to focus the attention only on samples characterized by the presence of WL at sufficiently high temperatures, useful to overcome the limitations related with used experimental setup. The results are shown in the following, together with a theoretical discussion, and confirm the hypothesis made. As a consequence, noise spectroscopy could represent a very sensitive tool to detect WL effects in different systems.

## Results

### dc transport and magnetic properties

**Figure 1:**
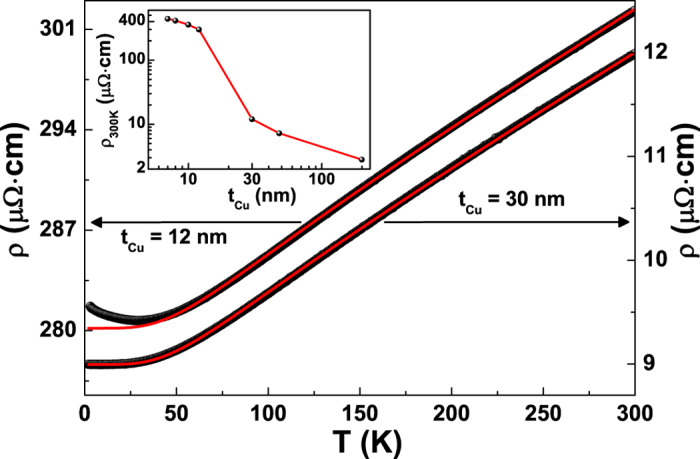
Resistivity versus temperature curves. Black dots refer to Cu thin films having thickness: *t*_*Cu*_ = 12 nm (left axis) and 30 nm (right axis). The red solid lines are the best fit to the measured data by the Bloch-Gruneisen formula, see equations (1) and (2) in the text. In the inset is shown the thickness dependence of the room temperature resistivity for the samples here investigated. The line is a guide to the eyes.

Below a thickness of 30 nm, the investigated thin copper films exhibit a change in their resistivity, as evident in the inset of Fig. 1 at room temperature. To better understand this feature, the temperature dependence of the resistivity of films with thickness *t*_*Cu*_ = 30 nm and *t*_*Cu*_ = 12 nm has been measured and shown in Fig. 1. The 30 nm sample is characterized by a metallic behaviour down to the lowest investigated temperature, while the sample of 12 nm shows a resistance minimum at temperature *T*_*min*_ = (28.0 ± 0.1) K and then the resistance rises as *T* → 0. This behaviour has been extensively studied and several mechanisms have been considered for its interpretation^5^. In particular, WL, electron-electron interactions, and the presence of Kondo impurities can be equally responsible for a resistivity upturn in the low-temperature limit. In this respect, the comparison of experimental results obtained with different techniques (such as standard dc characterizations, magnetic measurements, and noise spectroscopy) is necessary to identify the specific transport process of the charge carriers.

In nonmagnetic metals, the temperature dependence of the electrical resistivity arises mainly from the electron-phonon interaction, as described by the Bloch-Gruneisen formula^21^
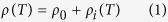
with
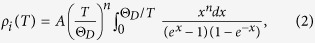
where *A* is a constant depending on the electrons velocity at the Fermi surface and their density in the metal; Θ_*D*_ is the Debye temperature; *ρ*_0_ is the residual resistivity, due to defects, impurities, size effects, and grain boundary scattering. In particular, it is assumed that all these scattering mechanisms contribute only to the elastic scattering rate, thus *ρ*_0_ ∝ 1/*τ*_0_. Conversely, the temperature-dependent contribution *ρ*_*i*_ (*T*) is related to electron-phonon and electron-electron interactions^22^. Moreover, the exponent *n* in equation (2) is usually fixed at 2, 3, or 5 depending on the nature of interaction: in the case of copper *n* = 5^23^,^24^.

**Figure 2:**
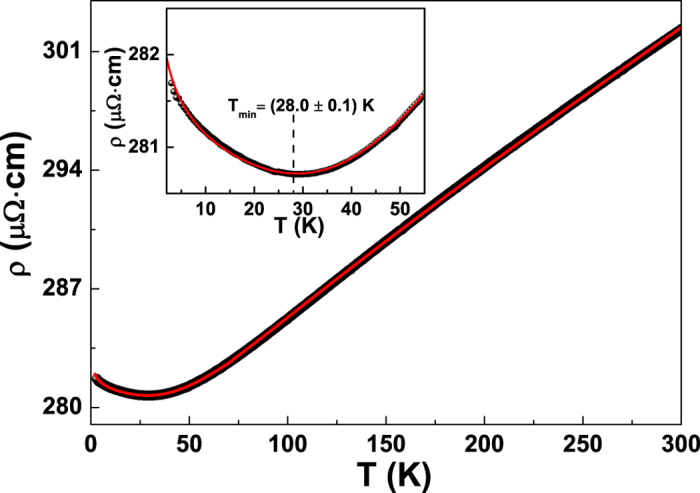
Resistivity data of the 12 nm sample. The red solid line is the best fit to the experimental data by using equations (3) and (4) including the WL correction, as reported in the text. The inset shows the low-temperature region along with the best fitting curve.

For thin disordered metallic films the Bloch-Gruneisen model is not able to reproduce the low-temperature resistivity behaviour, which is usually characterized by an upturn associated with the two-dimensional (2D) WL. This phenomenon is a well-known transport process for Cu ultrathin films, as widely reported in the scientific literature^1^, and produces a logarithmic quantum correction to the Drude conductivity (e.g., see the curve of the 12 nm thick sample in Fig. 1). WL contributions are expected to become significant at temperatures for which the elastic scattering rate 1/*τ*_0_ becomes larger than the inelastic dephasing rate 1/*τ*_Φ_, and the low-temperature correction to the normalized conductivity for a 2D film of thickness *t* can be expressed as
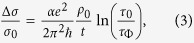
where *α* is a constant of the order of unity; *e* and *ħ* are the electron charge and reduced Planck constant, respectively; *τ*_Φ_ is the characteristic time of electron phase memory relaxation due to inelastic processes. Electron-phonon and electron-electron interactions produce a *τ*_Φ_ ∝ *T*^−*p*^, where *p* varies between 0.5 and 3^5^. By including this WL correction, the total resistivity *ρ*(*T*) can then be written as
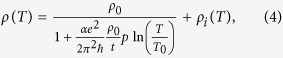
in which *T*_0_ identifies an upper limit of the WL onset. To evaluate the temperature dependence of *ρ*_*i*_, a best fit of *ρ*(*T*) as given by equations (1) and (2), with *n* = 5, to the experimental data has been performed and shown in Fig. 1 for both films (red solid lines). For the thicker film the Debye temperature Θ_*D*_ and the coefficient *A* are considered as free fitting parameters, obtaining the best values with Θ_*D*_ = (283 ± 1) K and *A* = (11.87 ± 0.02) *μ*Ω cm. For the sample showing the resistance minimum, the same fit is performed in the temperature range 50−300 K by considering also the residual resistivity *ρ*_0_ as a fitting parameter. The values *ρ*_0_ = (280.0 ± 0.5) *μ*Ω cm and *A* = (88.2 ± 0.1) *μ*Ω cm are now obtained, while Θ_*D*_ is found to be the same as for the 30 nm film, within the experimental error. By substituting into equations (3) and (4) all these parameters evaluated with the procedure previously described, it is possible to reproduce the *ρ* versus *T* behaviour of the thinner film by considering *αp* and *T*_0_ as free fitting coefficients. The best fitting curve, shown in Fig. 2 as red solid line, testifies a good agreement with the experimental data also in the low-temperature limit (see the inset to Fig. 2 for details), and is obtained with *αp* = (0.63 ± 0.05) and *T*_0_ = (68 ± 3) K. The value of *αp* is consistent with that reported in the literature for Cu ultrathin films^18^,^25^.

**Figure 3:**
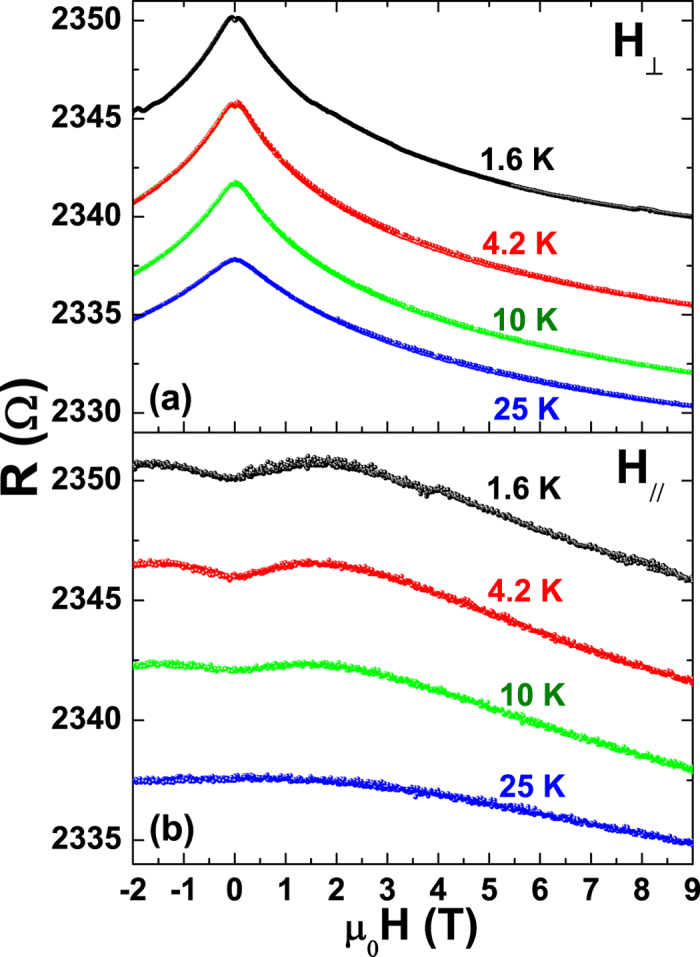
Magnetoresistance data of the 12 nm sample. The curves, showing the magnetic field dependence of the resistance in the temperature range 1.6−25 K, are vertically shifted by 1 Ω for clarity from top to bottom. (**a**) The magnetic field is applied perpendicular to the film surface. (**b**) The magnetic field is applied parallel to the film surface.

The results of the dc electrical characterization, alone, can not discriminate the specific transport process responsible for the low-temperature conduction in the disordered Cu ultrathin films, here investigated. To this purpose, resistance versus magnetic field measurements *R*(*H*) can give an effective proof of the existence of WL effects. Figure 3 shows the *R*(*H*) data for the 12 nm sample in the temperature range between 1.6 and 25 K for two field orientations, perpendicular *H*_⊥_ (a) and parallel *H*_//_ (b) to the film surface, with the bias current always perpendicular to the field. The measured magnetoresistance *MR* = [*R*(*H*) − *R*(0)]/*R*(0) is negative and shows large anisotropy according to the direction of the applied magnetic fields. The negative and anisotropic magnetoresistance has been extensively reported on thin copper films and well described within the WL theory. Conversely, for thicker films the standard metallic magneto-transport behaviour has been reported^18^,^26^. A positive magnetoresistance can be observed at low fields and below 10 K, and is usually ascribed to the presence of the spin-orbit coupling^27^.

### Noise properties

**Figure 4:**
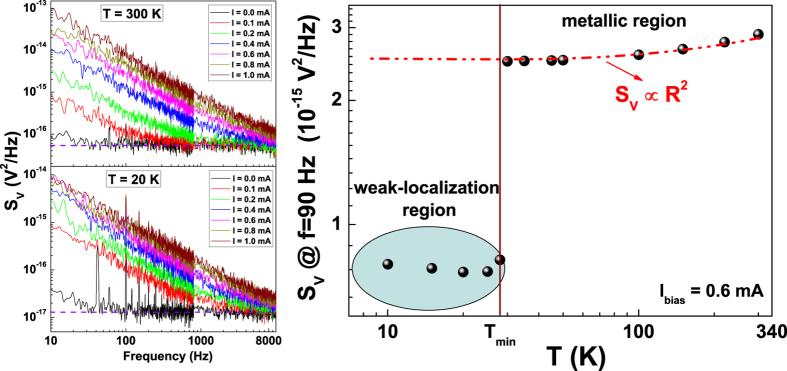
Voltage-noise experimental behaviour. The spectral traces above (*T* = 300 K) and below (*T* = 20 K) the crossover temperature *T*_*min*_ are shown in the left panel for the 12 nm sample. Its noise amplitude temperature dependence, at a reference frequency of 90 Hz and a fixed bias current level of 0.6 mA, is instead reported in the right panel. The red dashed line represents the best fitting curve to *S*_*V*_ by using the *R*^2^ experimental data.

In consideration of the strict connection between unusual 1/f electric noise and WL, reported for superconducting cuprates^11^ and manganite compounds^12^, a detailed voltage-spectral density analysis has also been performed on Cu ultrathin films. The low-frequency power spectral densities *S*_*V*_, measured at several temperatures and bias currents, are shown in Fig. 4 (left panel) for the 12 nm sample. When a current is supplied, the typical 1/f frequency dependence is clearly visible over a flat background spectrum, corresponding to the thermal Johnson noise and the electronic chain noise 1.4 × 10^−17 ^V^2^/Hz (for reference see the black traces in Fig. 4). Spurious contributions, due to external contact noise, are removed by using the experimental procedure described in ref. 28. The shape of *S*_*V*_ is the same above and below *T*_*min*_, as shown by the experimental data at 300 K and 20 K in Fig. 4. Conversely, the amplitude of the noise is different when the crossover between WL and metallic region occurs. This can be clearly observed in Fig. 4 (right panel), where the temperature dependence of *S*_*V*_, at a reference frequency of 90 Hz and fixed bias current of 0.6 mA, is shown as black dots. In these conditions, the standard theory of resistance fluctuations in metals predicts the direct proportionality *S*_*V*_ ∝ *R*^2^ 29. A good agreement between the experimental quantities is obtained by a fitting procedure only above *T*_*min*_ (see the red dashed curve in Fig. 4). This is not found below *T*_*min*_, where quantum interference effects produce an upturn of the resistivity. In particular, the WL region is characterized by unconventional features of the noise level not trivially related to resistance fluctuation processes, as at higher temperatures.

**Figure 5:**
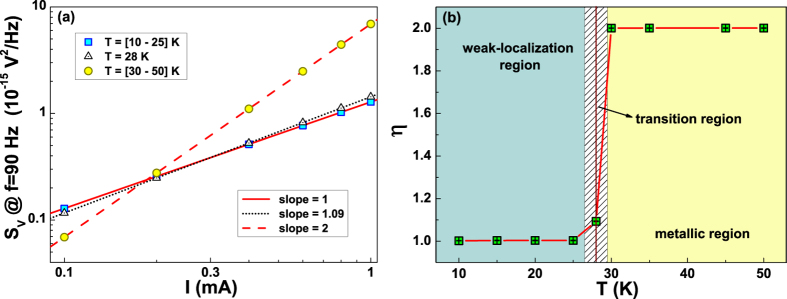
Noise behaviour as a function of the applied bias. The current dependence of the voltage-spectral density at *T* = 10 K (blue squares), at *T*_*min*_ = 28 K (open triangles), and at *T* = 50 K (yellow dots) is shown in the log-log graph of panel (a). The blue squares and the yellow dots are indicative of the *S*_*V*_ versus *I* slope in the 10–25 K and 30–50 K temperature ranges, respectively. The lines are the best fitting curves with equation (5). In panel (b), the temperature dependence of the power exponent *η* of equation (5) is shown around *T*_*min*_. The sample analyzed is the 12 nm, characterized by the WL regime.

A theoretical explanation of this behaviour has been given in ref. 12 by addressing the universal conductance fluctuation (UCF) mechanism as the source of 1/f noise in WL regime. In this context, a linear dependence of the 1/f noise on *I* at temperatures below *T*_*min*_ is expected. Conversely, above the crossover at *T* > *T*_*min*_ a standard quadratic current dependence of *S*_*V*_ is predicted^12^. The same analysis has been performed for the 12 nm Cu ultrathin film and the results are shown in Fig. 5(a). In this figure, it is evident a linear current dependence of the voltage-noise at low temperatures, in the WL regime, while at higher temperatures, in the normal conductance regime, although with comparable resistance values a quadratic bias dependence is found. This behaviour seems to be a universal feature of WL systems.

The bias dependence of the experimental data of Fig. 5(a) can be reproduced in terms of the power law
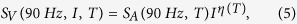
where *S*_*A*_ is the temperature-dependent amplitude of the spectral density at the reference frequency of 90 Hz; *η* is the power exponent. The best fitting curves by using equation (5) are also shown in Fig. 5(a). A good agreement between the functional form considered and the measured voltage-noise is obtained with values of *η* switching from 1.00 ± 0.01 in the WL region to 2.00 ± 0.01 in the metallic region. This can also be seen in the temperature dependence of *η*, shown in Fig. 5(b). Here, it is visible the presence of a region close to *T*_*min*_, where a sign of a transition appears. This behaviour suggests that a simple probe of WL can be done by means of noise spectroscopy, by looking at the transition from a linear to a quadratic current dependence of the 1/f spectral density. The reproducibility of the experimental evidence of Fig. 5(b) in different samples and materials (such as Cu, Nd_1.83_Ce_0.17_CuO_4+*δ*_, and La_0.7_Ba_0.3_MnO_3_ films reported here, in ref. 11 and in ref. 12, respectively) gives the clear indication for a common universal origin of the fluctuation processes of the charge carriers in WL regime.

## Discussion

The specific feature of the voltage-spectral density can be understood from the model described in ref. 12. Here, the main steps of the model derivation are briefly recalled. In WL the length *L*_Φ_, over which the diffusive motion of the electrons preserves the phase memory, is much longer than the electronic mean free path 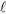. Consequently, quantum corrections to the classical motion become relevant and produce localization as the result of the destructive interference of time reversed loop trajectories triggered by a specific impurity configuration. The relic of the quantum nature of the electrons (i.e., the interference effects) manifests itself macroscopically with an increase of the sample resistance. However, the effect on the fluctuation properties of the system is much more pronounced, compared to the small change of the resistance. Indeed, the partial phase coherence of the sample below *T*_*min*_ makes effective the coupling between impurity motion and universal conductance fluctuations. The nonequilibrium dephasing mechanism affecting a coherent region of linear dimension *L*_Φ_ dresses the fluctuation mechanisms responsible for the conventional 1/f noise at high temperature (*T* > *T*_*min*_) and produces, below *T*_*min*_, a peculiar linear dependence on the bias current. Close to the crossover temperature *T*_*min*_, the voltage-spectral density *S*_*V*_ (*f*) depends on the variance 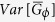 of the conductance of a single coherent subsystem averaged over the relevant transport energy window Δ*E* (i.e., 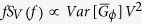). From elementary statistics considerations it follows (Δ*E* > *E*_*c*_)
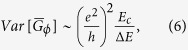
being 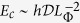 the Thouless energy. Equation (6) implies that the fluctuations of the energy averaged conductance 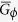 of a generic coherent region exhibit a reduced variance compared to the universal value (*e*^2^/*h*)^2^ as the effect of the incoherent contribution of several uncorrelated energy intervals of size *E*_*c*_ contained in Δ*E*. Following ref. 12, it is found that Δ*E* = *k*_*B*_*T* in the metallic region (*T* > *T*_*min*_), while Δ*E* = *eV*_Φ_ = *χeV* in the WL regime (*T* < *T*_*min*_). The former is the energy available to promote thermal excitation of electrons above the Fermi level, the latter is an energy scale induced by the average voltage drop *V*_Φ_ experienced by the generic coherent region. The dimensionless factor *χ* relates the macroscopic voltage drop across the sample *V* to *V*_Φ_, and contains statistical information on the topology of the random network explored by the current. For the simple topology given by the one dimensional random resistors network of length *L* biased by the voltage *V*, the average voltage drop experienced by a coherent region of length *L*_Φ_ is given by *v*_Φ_ = (*L*_Φ_/*L*)*V*, being *L*/*L*_Φ_ the number of coherent subsystems. An estimate of *V*_Φ_ in two dimensions can be obtained averaging the one dimensional result over a representative set of path lengths, i.e., 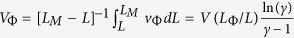 with *γ* = *L*_*M*_/*L* > 1. Increasing the temperature *T*, the energy scale *eV*_Φ_ ∝ *T*^−*p*/2^, with the same index *p* as in equation (4) depending on scattering process and dimensionality^5^, is progressively reduced making the thermal dephasing as the main inelastic mechanism (i.e., Δ*E* = *k*_*B*_*T*). In order to capture the above mentioned mechanisms using a minimal model suitable for a 2D random network, a temperature-independent parameter *χ* is introduced and Δ*E* ~ *max*[*eV*_Φ_, *k*_*B*_*T*] is assumed. By identifying *χ* with 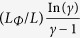, its temperature independence is recognized for *T* < *T*_*min*_ as induced by the low-temperature saturation of *L*_Φ_ to some constant value related to the geometric confinement and to the boundary effects. This provides an upper limit to the sample coherence.

**Figure 6:**
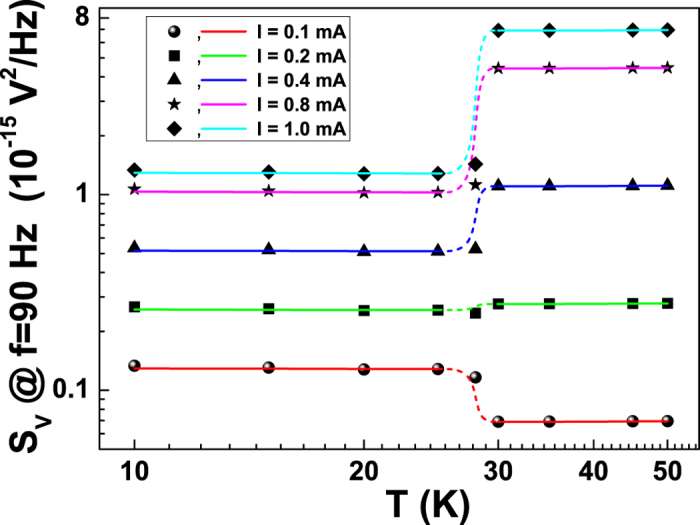
Temperature dependence of the voltage-spectral density at 90 Hz for different bias currents. The solid lines are computed by using equation (7), with 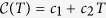 in the metallic region and 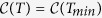 in the WL regime. The best fitting parameters are: *χ* = (5.55 ± 0.09) × 10^−3^, *c*_1_ = (1.1 ± 0.3) × 10^−2^, *c*_2_ = (3.23 ± 0.01) × 10^−2^ K^−1^.

This model of Δ*E* is just a crude approximation having the advantage of minimizing the number of free parameters of the theory, while it can not describe in detail the crossover region (*T* ≈ *T*_*min*_) where thermal and bias dephasing can produce combined effects. As a consequence, in WL regime the current flowing through the system provides a dephasing mechanism of the UCF with a characteristic dephasing time ~*ħ*/(*eV*_Φ_), while a thermal dephasing time ~*ħ*/(*k*_*B*_*T*) describes the high-temperature regime (*T* > *T*_*min*_). Equation (6) reproduces the asymptotic behaviour of the fluctuation mechanisms close to *T*_*min*_ and induces the following model for the voltage-spectral density^12^
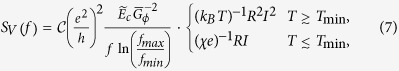
where the energy scale 
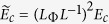
 has been introduced, while the proportionality factor 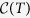 depends on the number of fluctuators coupled to the current within a typical coherent region. This number is proportional to 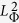, and saturates in the low-temperature limit to the constant value 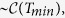 according with a freezing effect. In Fig. 6, equation (7) (solid lines) is compared with the experimental data (full symbols) and the relevant energetic scales involved in the nonequilibrium dephasing mechanisms affecting the coherence of the UCFs are extracted. Despite the limited number of free fitting parameters, an agreement is clearly evident above and below *T*_*min*_, while in the crossover region an interpolation line (dashed) is added just as a guide for the eyes.

The analysis of the energetic scales reconstructed by the fitting procedure of equation (7) (see in Supplementary Fig. 1) validates the dephasing model of UCFs. As expected, in the temperature range here considered, the applied bias represents a relevant dephasing source (*eV*_Φ_ > *E*_*c*_), under the specific condition that the energy window induced by the external currents is higher than the thermal energy *k*_*B*_*T* (see in Supplementary Fig. 1). These results have been already shown in oxide conductors^12^, but not yet reported in metals exhibiting quantum transport phenomenology. Such nonlinear effects of the voltage fluctautions have not been described in previous works^30^,^31^, because very small values of the bias currents were used. Moreover, differently from these old experimental findings on disordered simple metals, the noise data here presented cover nonequilibrium fluctuations across the whole metal-insulator transition. It is worth noting, that the temperature behaviour of the 1/f amplitude changes when the quadratic and linear bias dependencies are explored. In particular, from Fig. 6 it is clearly visible a low-temperature enhancement of the 1/f voltage-spectral density for small bias currents (e.g., see the red curve at *I* = 0.1 mA), as also reported in previous experiments^32^,^33^. Conversely, at large bias currents (e.g., see the curves at *I* ≥ 0.4 mA in Fig. 6) an opposite effect on the 1/f component is observed. These evidences give a strong indication of the crucial role played by the bias current in quantum transport phenomena.

In order to further characterize the occurrence of WL, the typical lengths involved in the WL regime have been analyzed. The coherence length *L*_Φ_ and the spin-orbit length *L*_*so*_, that estimates the intensity of spin-orbit coupling, can be experimentally extracted from magnetoresistance measurements. In particular, the magnetic field dependence of the correction to the conductance in two dimensions is expressed as^34^
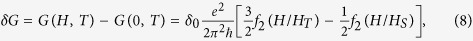
where the function *f*_2_(*x*) is given by *f*_2_(*x*) = ln (*x*) + Ψ(1/2 + 1/*x*) with Ψ as the digamma function, and *δ*_0_ is a proportionality factor. The characteristics fields *H*_*T*_ and *H*_*S*_ are the triplet and singlet fields, defined, in absence of magnetic impurities, as
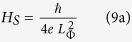

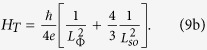


**Figure 7:**
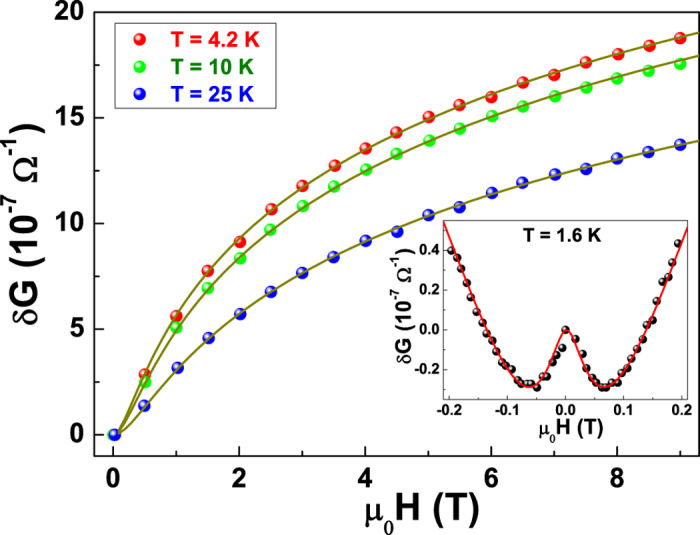
Magnetic field dependence of the correction to the conductance. The experimental data points and the best fitting curves with equation (8) (solid lines) are shown for the temperatures of 4.2, 10, and 25 K. The presence of a spin-orbit effect is clearly visible only at the lowest investigated temperature of 1.6 K and is shown in the inset.

**Figure 8:**
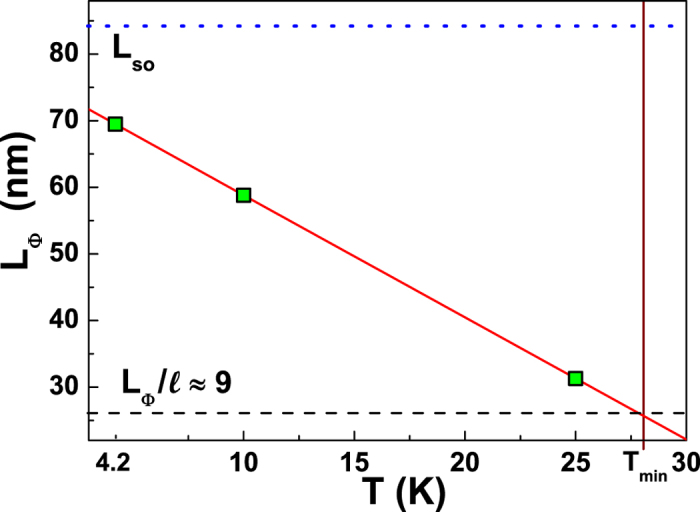
Temperature dependence of the the coherence length *L*_Φ_. The experimental data are shown as green squares. The horizontal lines represent: the length *L*_*so*_ = (84 ± 2) nm (dotted), and 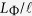 ratio at *T*_*min*_ (dashed). The red solid line is only a guide to the eyes.

The experimental data can be modelled with equation (8), allowing the extraction of the characteristic lengths from equations (9). More in detail, the best fitting curve of the magnetoresistance at the lowest investigated temperature of 1.6 K (see inset of Fig. 7) gives *L*_*so*_ = (91 ± 2) nm. On the other hand, in the temperature range from 4.2 to 25 K, *L*_*so*_ takes a temperature-independent value of (84 ± 2) nm, in accordance with the Fermi-liquid theory^35^. By fixing the spin-orbit length to this constant value, it is possible to evaluate *L*_Φ_ for the same temperatures of Fig. 3. The agreement between the theoretical prediction and the data points is clearly visible in Fig. 7. From this fitting procedure, the low-temperature behaviour of *L*_Φ_ can be obtained and is shown in Fig. 8 as green squares. Here, it is deduced that below *T*_*min*_ the effect of the spin-orbit coupling is negligible and equation (8) reduces to the 2D WL formula (*L*_Φ_ < *L*_*so*_). Conversely, in the low-temperature regime (*T* < 4.2 K) *L*_Φ_ becomes comparable with *L*_*so*_, inducing antilocalization corrections in the low-field magnetoresistance curve (see inset of Fig. 7).

The WL regime requires 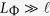. In order to verify this condition, the mean free path can be computed from the sample resistivity according to ref. 36. A value of 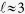 nm has been extracted for the 12 nm Cu film, in a temperature range close to *T*_*min*_. This estimation (see Fig. 8 for details) implies that the WL transition occurs when 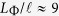. A distance of 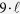 is sufficient to enable closed electronic (phase coherent) trajectories, which link several grain boundaries (4 in average). It is worth mentioning here, that ultrathin Cu films manifest the tendency to present a system growth based on coalescence of mesoscopic grains, whose linear dimensions increase with the thickness. Therefore, the resistivity of ultrathin films characterized by a microscopic granular structure is generally much higher than the resistivity of a thicker sample, this critical thickness being of the order of 40 nm. This is confirmed by the inset to Fig. 1, where the sample with *t*_*Cu*_ = 12 nm shows a resistivity value of two orders of magnitude higher than the thickest film (*t*_*Cu*_ = 200 nm), having a bulk-like behaviour. Assuming a grain boundary limited mean free path, an average grain diameter of ∼6 nm can be deduced for the 12 nm Cu film. This value is in agreement with the resistivity of the investigated samples, while for more conducting films a grain size of the order of the thickness is expected. Such evaluation is also compatible with the assumption of a grain size about 15−20 nm, as reported in ref. 37, by considering an additional intragrain scattering mechanism which limits the mean free path.

In conclusion, several Cu ultrathin films of different thickness have been fabricated and electrically characterized. The sample of thickness *t*_*Cu*_ = 12 nm shows an upturn of the resistivity below the crossover temperature *T*_*min*_ ≈ 28 K. The analysis of the resistivity and the characteristic negative magnetoresistance indicates that a localized regime is established at low temperature. The signature of the spin-orbit interaction has been also detected in the low-field magnetic response of the sample. Once a weak-localization regime (*T* < *T*_*min*_) has been confirmed, detailed voltage-spectral density analysis has been performed. While the high-temperature (*T* > *T*_*min*_) behaviour of *S*_*V*_ versus *I* takes the usual quadratic form, in the WL regime an anomalous linear dependence on the applied bias is detected. These findings present the same distinctive features described in ref. 12. In particular, in the WL regime, the voltage fluctuations are strongly affected by the temporal variation of the UCFs, being the latter a coherence effect. The variance of these fluctuations is reduced due to the dephasing effect of the bias current *I*. As a consequence, it has been confirmed, also in the case of a simple metallic thin film, that the anomalous linear dependence of *S*_*V*_ versus *I* is due to the dephasing effect on the local conductance fluctuations of the current bias exploring the percolative network of the sample. Thus, the anomalous fluctuation behaviour detected in the WL regime seems to be a material-independent feature only associated with partial restoration (and its local destruction by the measuring action of the current) of the sample coherence. Once the anomalous *S*_*V*_ behaviour has been unambiguously associated with the WL regime, it will also provide a simple diagnose of localization effects in innovative two-dimensional systems like graphene, topological insulators, and two-dimensional electron gases. These materials represent the technological platform to develop topological computation (for instance, based on Majorana modes), which requires a fixed magnetic bias for the stabilization of the device working point. In this case, magnetoresistance measurements are difficult to be performed and a purely electric probe of WL mechanisms, such as noise spectroscopy, could be very useful.

## Methods

Ultrathin Cu films were deposited on Si(100) substrates covered by 200 nm thick SiO_2_ in ultra-high-vacuum by dc magnetron sputtering. The system is equipped with a movable sample holder which allows to fabricate different samples in the same run under the same deposition conditions.

The samples were structured in a standard Hall geometry of length *L* = 1 mm and width *W* = 100 *μ*m allowing four contact electrical measurements. The pattern was realized on the SiO_2_/Si substrates using standard optical lithography and lift-off procedure. Subsequently, the films were deposited in a base pressure in the high 10^−8^ mbar range and a sputtering argon pressure of 5.5 × 10^−3^ mbar at typical rates of 0.29 nm/s, controlled by a quartz crystal monitor, calibrated with low-angle X-Ray reflectivity measurements.

Seven different films were prepared with thickness *t*_*Cu*_ = 7, 8, 10, 12, 30, 48, 200 nm. To prevent the surface degradation or oxidation of the active area between electrical pads, a 10 nm thick insulating cap layer of SiO_2_ was selectively rf sputtered on the structures just after lift-off using a metallic shadow mask. The electrical contacts were made through rf sputtered 50 nm thick Au pads defined by optical lithography and lift-off. The thickness dependence of the room temperature resistivities *ρ*_300*K*_, shown in the inset of Fig. 1, reminds the ones reported in the literature for high quality Cu films^38^,^39^. Concerning the resistivity values, it is useful to compare them with the values of the sheet resistance of island-like thin Cu films deposited by the same technique and in similar depositions conditions^37^. For the thinnest films here investigated, the sheet resistance, *R*_*s*_ = *ρ*_300*K*_/*t*_*Cu*_, spans from *R*_*s*_ = 250 Ω/□ for *t*_*Cu*_ = 12 nm to *R*_*s*_ = 330 Ω/□ for *t*_*Cu*_ = 8 nm, whereas in ref. 37 for a 5-nm film it is *R*_*s*_ ≈ 800 Ω/□. This result indicates that the samples under study have a more continuous morphology and that the crucial role in the scattering process seems to be played by the scattering at grain boundaries.

The dc electric transport and magnetoresistance measurements were carried out in a Cryogen Free Magnet system by Cryogenic Ltd., equipped with an integrated cryogen-free variable temperature insert (operating range between 1.6 and 300 K) and a superconducting magnet able to generate a magnetic field up to 9 T. In this system, the samples were biased with a fixed current of 10 *μ*A by using a Keithley 2430 Sourcemeter and the output voltage was recorded with a Keithley 2182 Nanovoltmeter. Electric noise measurements, instead, were performed by using a closed-cycle refrigerator, operating in the 8- to 325-K range. The temperature stabilization, realized through a computer-controlled feedback loop, was better than 0.1 K. The sample temperature was measured with a Cernox resistor thermometer, in contact with the sample holder. A low-noise Keithley dc current source was used for the biasing of the samples. The dc voltage drop was measured with a digital multimeter, while the ac voltage signal was amplified with a low-noise PAR5113 Preamplifier and analyzed by a dynamic signal analyzer HP35670A.

## Additional Information

**How to cite this article**: Barone, C. *et al*. Nonequilibrium fluctuations as a distinctive feature of weak localization. *Sci. Rep*. **5**, 10705; doi: 10.1038/srep10705 (2015).

## Supplementary Material

Supplementary Information
