# Supplementary material for: Nonequilibrium fluctuations as a distinctive feature of weak localization
Source: Sci Rep. 2015 May 29;5:10705. doi: 10.1038/srep10705 (PMC4448654; doi:10.1038/srep10705)
Supplement: Supplementary Information [file srep10705-s1.pdf]

Supplementary Information for "Nonequilibrium fluctuations as a distinctive feature of weak localization"

C. Barone[1,2],* F. Romeo[1,2], S. Pagano[1,2], C. Attanasio[1,2], G. Carapella[1,2], C. Cirillo[1,2], A. Galdi[2,3], G. Grimaldi[2], A. Guarino[1,2], A. Leo[1,2], A. Nigro[1,2], and P. Sabatino[1,2]

[1]*Dipartimento di Fisica "E.R. Caianiello", Università di Salerno, I-84084 Fisciano, Salerno, Italy*

[2]*CNR-SPIN, UOS di Salerno, I-84084 Fisciano, Salerno, Italy*

[3]*Dipartimento di Ingegneria dell'Informazione, Ingegneria Elettrica e Matematica Applicata, Università di Salerno, I-84084 Fisciano, Salerno, Italy*

*Electronic address: cbarone@unisa.it

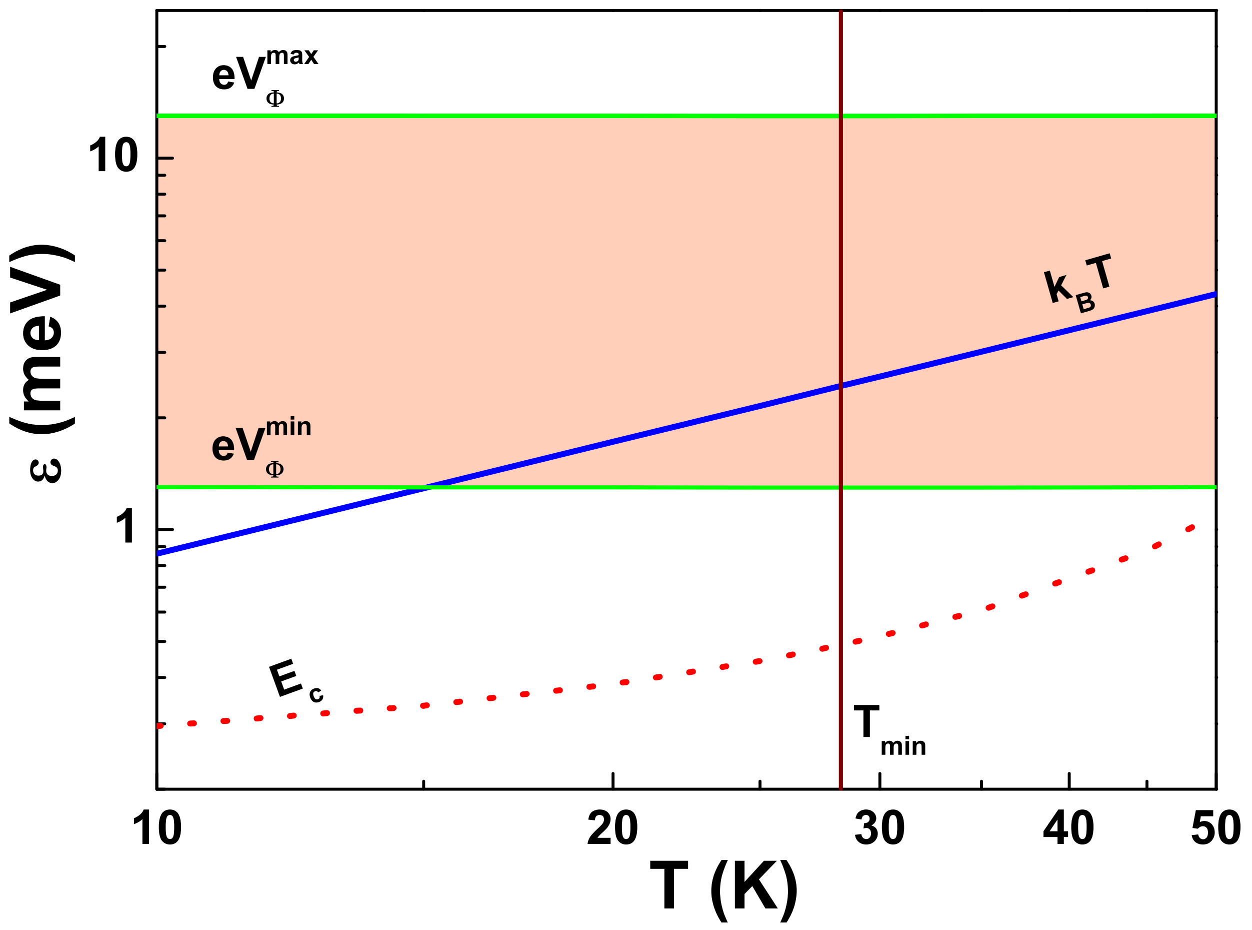

FIG. 1: **Energetic scales involved in the electronic transport for the 12 nm Cu sample.** Solid blue line represents the thermal energy $k_B T$, while dotted red curve is the Thouless energy E_c. The bias energies eV_Φ^{min} and eV_Φ^{max} are computed by the fitting procedure of equation (7), as specified in the main text, and by using $I = 0.1$ mA and $I = 1.0$ mA, respectively. T_{min} is the temperature corresponding to the resistivity minimum.
